# Pyrethroid Acaricide Resistance Is Proportional to P-450 Cytochrome Oxidase Expression in the Cattle Tick* Rhipicephalus microplus*

**DOI:** 10.1155/2018/8292465

**Published:** 2018-07-04

**Authors:** Raquel Cossío-Bayúgar, Francisco Martínez-Ibañez, Hugo Aguilar-Díaz, Estefan Miranda-Miranda

**Affiliations:** ^1^Centro Nacional de Investigación Disciplinaria en Parasitología Veterinaria, Instituto Nacional de Investigaciones Forestales Agrícolas y Pecuarias, Jiutepec Morelos, Mexico; ^2^Departamento de Ectoparásitos y Dípteros, Servicio Nacional de Sanidad, Inocuidad y Calidad Agroalimentaria SAGARPA, Jiutepec Morelos, Mexico

## Abstract

The goal of the present study was to assess the gene expression of xenobiotic metabolizing enzymes (XMEs) Cytochrome P-450 (CYP) and carboxylesterase (CE) related to detoxification of synthetic pyrethroids, plus acetylcholinesterase (AChE), in field isolates of acaricide-resistant Rhipicephalus microplus. The XMEs expression levels were assessed by mRNA measurement using quantitative reverse transcription PCR. The XME expression levels of field-isolated acaricide-resistant ticks were compared against acaricide-susceptible reference ticks used in this study as a gene expression baseline and represented as relative expression units (REU). Field isolates were subjected to toxicological bioassays and determined resistant to all the Pyr acaricides (Pyr), whereas most of them were found susceptible to organophosphorous acaricides (OP), with the exception of three isolates, which exhibited moderate resistance to Diazinon. Significantly higher levels of CYP were detected in pyrethroid-resistance ticks when compared to Su ticks (P<0.01). A linear regression analysis showed that pyrethroid acaricide resistance levels of R. microplus were proportional to the CYP expression levels (correlation coefficient (R):0.85; P<0.05). Analysis on CE expression levels showed only one isolate resistant to Pyr and OP with a statistically significant increase (P<0.01). AChE expression levels showed statistically significant (P<0.01) subexpression in all tick isolates when compared to the susceptible reference. Our results suggest that pyrethroid acaricide resistance in the cattle tick may be diagnosed by measuring the CYP expression levels using quantitative PCR.

## 1. Introduction

Pesticide resistance in arthropods is a multifactorial phenomenon involving behavioral, biochemical, and metabolic mechanisms designed to counteract the effect of the pesticide on the target organism [1, 2]. Previous studies have shown that pesticide resistance is facilitated by the action of xenobiotic metabolizing enzymes (XME) [3]. XME are found in most metazoan organisms, and they provide an enzymatic mechanism of defense against the potentially toxic actions of natural xenobiotics compounds [4]; these enzymes act by biochemically transforming exogenous and endogenous chemical radicals into hydrophilic derivatives through reactions collectively known as biotransformation [3].

The cytochrome P-450 monooxygenase (CYP) enzymatic family is a set of ubiquitous enzymes that participate in the regulation of endogenous bioactive molecules, such as hormones; they control the metabolism and detoxification of cell damaging chemicals such as plant toxins, drugs, and pesticides in a large variety of arthropods [5]. As a consequence, arthropods with naturally high levels of CYP are insensitive to certain pyrethroid (Pyr) formulations, rendering these pesticides ineffective for certain types of pest control [6, 7]. CYP-mediated metabolism is implicated in the deactivation of pesticides in several pesticide-resistant arthropods, including* Culex* spp. [4],* Anopheles gambiae* [8],* Blattella germanica* [9],* Helicoverpa armigera* [10],* Tribolium castaneum* [11], and the house fly* Musca* spp. [12, 13]. Previous reports suggest that Pyr acaricide toxicity is neutralized in* R. microplus* through the metabolic action of CYP [14]. Another study reported high expression levels of CYP in Pyr acaricide-resistant ticks combined with subexpressed CYP levels in ticks that were resistant to organophosphorous (OP) based acaricides, suggesting that CYP expression is selectively modulated in response to different pesticide formulas [1, 15]. OP acaricides are chemically designed to be metabolized by the CYP enzymatic pathway in order to undergo transformation to a more toxic form [3]. As such,* R. microplus* tolerance against the OP acaricides Coumaphos and Diazinon has been linked to lower expression and/or enzymatic function of CYP. Loss of CYP expression in this case allows the ticks to block the metabolic transformation of the acaricide to a toxic molecule [3, 15]. Acetylcholinesterase (AChE) is found in a wide range of organisms, including arthropods; it regulates cholinergic transmission by hydrolyzing the neurotransmitter acetylcholine. Organophosphorous pesticides inactivate AChE by phosphorylation of the enzyme's active site [3]. An altered AChE able to resist phosphorylation of the active site produced by OP pesticides is a common resistance mechanism, used by* Musca domestica* [16],* Culex pipiens* [17],* Anopheles albimanus* [18], and* Drosophila* sp. [19]. However, an alternate mechanism may be to increase the expression of putative AChE enzymes that are responsible for sequestering and detoxifying the excess OP chemicals [20]. Members of the carboxylesterase (CE) family are closely related to AChE. Like AChE, CE also enhances pesticide detoxification in several pesticide-resistant arthropods via ester-hydrolysis and metabolism of xenobiotic compound [20–22]; both enzymes share a common affinity for several synthetic substrates [23]. Previous reports have implicated CE in the pesticide resistance of a large variety of pest arthropods, including the sheep ectoparasite* Lucilia cuprina* [24, 25] the human blood-sucking mosquitoes* Culex* sp.,* Aedes* sp., and* Anopheles* sp. [21, 25] and the peach-potato aphid* Myzuz persicae* [26, 27]. Some of the acaricide resistance of the cattle tick is related to detoxification of synthetic pyrethroids mediated by ester bond hydrolysis of the acaricide by a specific CE [28]. Further studies have also shown that this CE gene expression and enzymatic activity are increased in Mexican pyrethroid-resistant strains of* R. microplus* ticks [29, 30] and a correlation between CE activity and cattle tick acaricide resistance [29–32]; additionally a study on Australian* R. microplus* ticks suggested that altered AChE and CE may work simultaneously during OP and Pyr acaricide resistance [20].

## 2. Materials and Methods

All animals used in these experiments were housed at the Isolation Units of the Centro Nacional de Investigación Disciplinaria en Parasitología Veterinaria at Instituto Nacional de Investigaciones Forestales, Agrícolas y Pecuarias (CENID-PAVET, INIFAP). This study was approved by the INIFAP Animal Experimentation and Ethics Committee and conducted considering ethic and methodological aspects in agreement with the Mexican regulations related to use, housing, and transport of experimental animals NOM-0 NOM-062-ZOO-1999.

### 2.1. Ticks

An acaricide-susceptible reference strain (Su) was used to establish a baseline for CYP, AChE, and CE gene expression (Table 1). The field-isolated ticks used in this work were obtained from cattle in the southern Mexican state of Michoacán during cattle inspection for the acaricide resistance-monitoring program mandated by the Mexican Federal Government. All the ticks were maintained and analyzed by bioassays at the Mexican FAO Regional Reference Laboratories for Diagnosis and Monitoring of Acaricide Resistance and other parasites of veterinary importance, at the Centro Nacional de Parasitología Animal (CENAPA, SAGARPA), Departamento de Ectoparásitos y Dípteros del Servicio Nacional de Sanidad, Inocuidad y Calidad Agroalimentaria (SENASICA-SAGARPA).

All the ticks used in this study were cultured as described previously [33]; cattle was infested with 2 X 10^4^ 10-15-day-old larvae. The engorged females were collected 21 days after the infestation, groups of ten engorged ticks per strain were then incubated in Petri dishes at 28°C, and 80% relative moisture until oviposition was completed as previously described [2]. The tick egg masses were collected and weighed, and 200 mg of the egg mass was aliquoted into vials until larval eclosion. The larvae were then kept at 28°C in 80% relative moisture.

### 2.2. Bioassays

Reference Su strain of ticks and tick isolates were assayed for Pyr and OP resistance by the acaricide discriminant doses bioassay [34]. Bioassays were run using acaricides diluted in trichloroethylene at the following concentrations: coumaphos 0.2%, diazinon 0.08%, chlorpyrifos 0.2%, cypermethrin 0.05%, deltamethrin 0.09%, and flumethrin 0.01%. One milliliter of each dilution was applied evenly to a 7 by 9 cm piece of filter paper. The trichloroethylene was allowed to evaporate from the filter paper for two hours. Treated papers were then folded in half and sealed onto slides with clips, thus forming a packet into which approximately one hundred 8-10 days-old larvae could be placed. The top of the packet was then sealed with another clip. The packets were kept at 27°C with 92% relative humidity for 24 h. The packets were then removed from the incubator and opened, live and dead larvae were counted, and the data were processed as survival rates (%) for each tick group and each acaricide concentration (Table 1).

### 2.3. Relative Quantification of Cytochrome P-450, Carboxylesterases, and Cholinesterases

The total RNA was isolated from each* R. microplus* tick strains and/or isolates following the manufacturer's instructions (RNAqueous®-4PCR kit, Ambion, TX, USA). The RNA was reverse transcribed to cDNA using random decamer primers as per the manufacturer's instructions (High Capacity cDNA Reverse Transcription Kit, Applied Biosystems).

The relative gene expression of CYP, CE, and AChE was quantified by real-time PCR as previously described (Cossio-Bayugar et al. 2009), using a fluorogenic 5' nuclease assay (TaqMan® system) and an ABI Prism 7300 Sequence Detector (Applied Biosystems, CA, USA). The TaqMan probes were designed based on reported DNA sequences for CE (GenBank accession: AF182283; AF286096, DQ533868), AChE (GenBank accession: AJ278345, AJ278344, AJ278343, AJ278342, and AF067771), and CYP (GenBank accession: AAD54000). The real-time PCR reactions included 900 nM of each primer, 300 nM of probe, and 1X TaqMan Universal Master Mix (Applied Biosystems). The real-time PCR cycling program consisted of one cycle at 50°C for 2 min and one cycle at 95°C for 10 min, followed by 40 cycles of 95°C for 15 s and 60°C for 1 min. The AChE, CE, and CYP gene-specific PCR primers and the TaqMan probes labeled with 6-carboxyfluorescein (FAM)/MGB were designed as follows: AChE forward primer AChEFor 5'- GGCACTGAAATGGATCCAGGAA-3', AChE reverse primer AChERev 5'- CGTGACTTCACCAGGGTTACC -3' and AChE TaqMan probe 5'-CCAAATGCAGCAATGTT-3'; CE forward primer CEFor 3'-CGACGCATTCCTTCCAAAGATG-5', CE reverse primer CERev 3'- TCGACGGACGCGAAGAAG-5' and CE TaqMan probe -CACTTGTAGCCATGAATC-5'; CYP forward primer CYPF 5'- CAAGCTGGTTGCTCTACATTATCGA -3', CYP reverse primer CYPR 5'- TTGGCCTCAGGACGAGTTC -3' and CYP TaqMan probe 5'- CCATGACATGAATCTTG -3'. The R. microplus eukaryotic 18S rRNA (Applied Biosystems, CA, USA) probe served as an endogenous control and was labeled with MGB®. To verify reproducibility, the real-time PCR analysis, including the 18S rRNA endogenous control, was independently performed twice with four replicates per experiment (n=8).

The RNA transcript levels for each strain and field isolates were determined by the 7300 SDS Software v1.2.2 * *(Applied Biosystems, CA, USA) using the comparative Ct method and the ΔΔ Ct method used to calculate the fold differences in CE, AchE, and CYP between samples; the Su expression levels were used as calibrator and named as relative expression units (REU), as described in the ABI Prism 7300 Sequence Detector real-time thermal cycler manufacturer's manual (Applied Biosystems, CA, USA), available at http://www3.appliedbiosystems.com/cms/groups/mcb_support/documents/generaldocuments/cms_042380.pdf.

### 2.4. Statistical Analysis

To find field isolates exhibiting significant expression levels, means and dispersion measurements of the relative gene expression of CE, AChE, and CYP from tick field isolates were compared against Su reference strain using ANOVA. Data set found to have significant differences of relative expression were analyzed by Tukey's HSD post-ANOVA test. All statistical tests were done using Prism 5 software (GraphPad Software Inc. CA, USA). Linear regression analysis was carried out using Prism 5 software (GraphPad Software Inc. CA, USA).

## 3. Results

The acaricide-susceptible reference strain exhibited a 0% survival rate when exposed to all the acaricides used in this study (Table 1).

Field isolates were resistant to all the Pyr acaricides used in this study, and most of the isolates were susceptible to the OP acaricides, with the exception of isolates 32, 38, and 45, which exhibited moderate resistance to Diazinon (Table 1). A summary of the survival rate of every isolate in response to each acaricide formula during the bioassays is displayed on Table 1.

**Figure 1:**
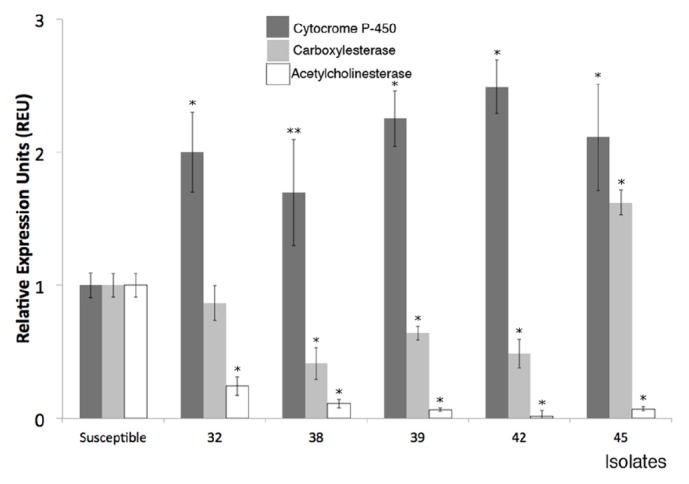
Cytochrome P-450 (CYP), carboxylesterase (CE), and acetylcholinesterase (AChE) gene expression by real-time PCR in acaricide-susceptible ticks and field isolates. The black bars represent the CYP data, the gray bars represent the CE data, and the clear bars represent the AChE data. The data are represented as relative expression units (REU) compared to the susceptible reference strain. The means and SD are shown. *∗* Statistically significant difference (P<0.01) compared to Su reference strain. *∗∗* Statistically significant difference (P<0.05) compared to Su reference strain.

The CYP, CE, and AChE gene expression levels of the Su reference strain were determined as to 1 relative expression unit (REU). All Pyr acaricide-resistant ticks exhibited a statistically significant overexpression of CYP, when compared against Su ranging from 1.69 to 2.49 REUs (Figure 1). Isolate number 45 exhibited double resistance, a statistically significant overexpression of CE of 1.62 REUs. CE subexpression was observed in the remaining tick isolates. AChE gene presented statistically significant subexpression in all tick isolates. The REUs for each isolate and each enzyme have been graphically represented in Figure 1.

**Figure 2:**
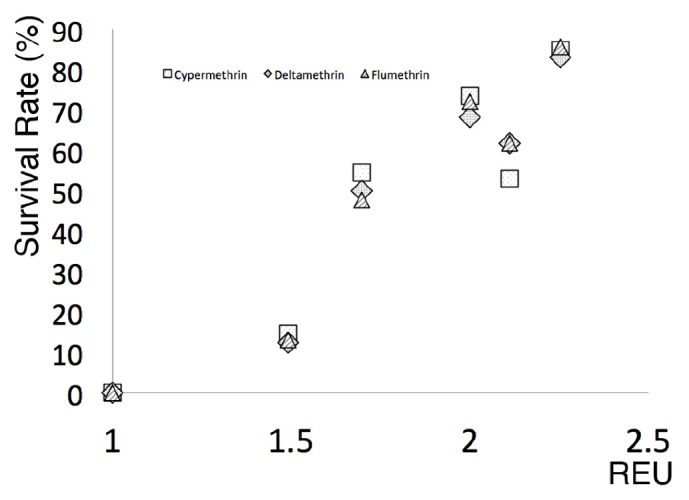
Linear regression analysis of acaricide-susceptible ticks and field isolates. The X axis represents the CYP expression in REUs and the Y axis represents the tick survival rate under different Pyr acaricide formulations; R of 0.85 (P<0.05).

The regression analysis of CYP REUs expression against the survival rate of Su reference strain field tick isolates exposed to Pyr acaricides showed a correlation coefficient (R) of 0.85 (P<0.05) (Figure 2).

## 4. Discussion

The ability of arthropods to exhibit resistance against pesticides is due to underlying genetic mechanisms that may involve the expression of one or several XMEs genes [35], which allow for increased enzymatic detoxification [32]. In this study, we analyzed the gene expression levels of CE related to detoxification of synthetic pyrethroids, AChE, and CYP from tick isolates with differing levels of acaricide resistance. Our results show that the expression patterns of these genes correlate with the toxicological profiles of the ticks, which strongly suggest a relationship between the XMEs gene expression and the acaricide resistance levels (Table 1, Figure 1). The high CYP expression levels corresponded with increased levels of resistance to all pyrethroid formulations; however, CYP overexpression did not promote resistance to other acaricides (Figure 1, Table 1). These results are consistent with the notion that CYP-mediated detoxification confers Pyr resistance by neutralizing Pyr acaricides [6, 12]. Accordingly, Pyr acaricide-resistant ticks showed a consistent enzyme expression profile with statistically significant overexpression of CYP for all isolates (P<0.01-0.05). A linear regression analysis of CYP expression against the Pyr acaricide survival rate of field-isolated ticks revealed that Pyr resistance was proportional to CYP expression, suggesting that monooxygenase XMEs may be neutralizing the Pyr acaricides, as occurs in the cattle tick and other Pyr-resistant pest arthropods [13, 14]. Our experimental data reinforces the role of CYP in the phenomenon of cattle tick acaricide resistance and they suggest that increased CYP expression leads to Pyr-resistant ticks. During this study we measured* R. microplus* expression levels of CE, which previous research had related to detoxification of synthetic pyrethroids and increased gene expression in Mexican pyrethroid-resistant strains of* R. microplus* ticks [29]. Our study on pyrethroid-resistant field isolates showed that only isolate 45 exhibited an increased CE and CYP gene expression, and toxicological bioassays demonstrated that isolate 45 was resistant to the OP Diazinon and all Pyr acaricides, suggesting a link between CE and Pyr and multiple acaricide resistance, corroborating previous studies [30, 31]. Most notably, all isolates showed significantly increased CYP expression (Figure 1) strongly suggesting that the principal Pyr resistance mechanism is mediated by cytochrome oxidases. AChE gene showed a statistically significant subexpression in all the analyzed isolates (Figure 1), reduced AChE expression may be explained by a lack of relationship between this enzyme and pyrethroid resistance on the field isolates analyzed; this result is consistent with other models of pesticide-resistant arthropods which show a strong correlation between high levels of CYP and the pesticide resistance phenomenon.

Our results also suggest that a measurement of CYP and CE gene expression by real-time PCR may be able to predict Pyr acaricide resistance. This hypothesis requires validation in field surveys and acaricide resistance field monitoring in order to establish a definitive trend and biochemical relationship between acaricide resistance and monooxygenase expression levels in the cattle tick. Further studies are needed to establish a diagnostic predictive parameter that will be useful for the management of pesticide resistance.

## Figures and Tables

**Table 1:** Survival rates (%) of susceptible reference strain and field isolates determined by acaricide bioassay.

	Organophosphates			Pyrethroids		
**Tick Sample**	Chlorpyrifos	Coumaphos	Diazinon	Cypermethrin	Deltamethrin	Flumethrin

**Su**	0	0	0	0	0	0

**Isolate** **32**	0	0	14.47	73.52	68.12	72.09

**Isolate** **38**	0	0	8.58	54.5	50	47.73

**Isolate** **39**	0	0	0	84.73	82.99	85.63

**Isolate** **42**	0	0	0	14.56	12.35	12.98

**Isolate** **45**	0	0	32.56	53.02	61.83	61.82
